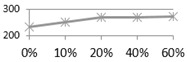# Acid-Alkali Resistance of New Reclaimed Tiles Containing Sewage Sludge Ash and Waste Glass

**DOI:** 10.3390/ma9070546

**Published:** 2016-07-07

**Authors:** Deng-Fong Lin, Kuo-Liang Lin, Huan-Lin Luo, Jia-Qin Xu

**Affiliations:** Department of Civil and Ecological Engineering, I-Shou University, Kaohsiung 84001, Taiwan; dflin@isu.edu.tw (D.-F.L.); hlluo@isu.edu.tw (H.-L.L.); chloelv111@gmail.com (J.-Q.X.)

**Keywords:** reclaimed tiles, sewage sludge ash, waste glass, acid-alkali resistance

## Abstract

In this study, properties of newly developed reclaimed tiles in a harmful environment were investigated. A portion of clay used to manufacture tiles was replaced with sewage sludge ash (SSA) and waste glass to produce the new reclaimed tiles. To investigate the effects of SSA and waste glass on the properties of the tiles, different specimens were blended and placed in acid-alkali solutions. The reclaimed tile specimens were manufactured by clay, 10% SSA, and five different mixes of waste glass replacement, namely, 0%, 10%, 20%, 40%, and 60%. These specimens were calcined at 1000 °C and subsequently underwent a series of tests, including TGA/DTA (thermogravimetric analysis/differential thermal analysis), SEM (scanning electron microscopy), XRD (X-ray diffraction), bending strength, weight loss, and porosity. Test results show that shortcomings associated with the introduction of the sludge ash were improved by the admixture of waste glass, especially in the aspects of shrinkage and bending strength. The study showed that the new reclaimed tiles performed relatively well in acid-alkali resistance tests but appeared to have better alkali resistance than acid resistance. It was also found that the optimal mix of such reclaimed tiles was 10% SSA, 10% waste glass, and 80% clay.

## 1. Introduction

Sewage sludge is the waste material that is produced during the treatment of industrial or municipal wastewater. As the amount of sludge increased with the implementation of environmental programs in many countries, handling of sewage sludge began to attract considerable attention. In agriculture, treated sewage sludge is used as biosolids to spread over land for non-food agriculture or food agriculture. However, this practice raises many disputes due to health and environmental concerns [1].

Containing SiO_2_ and Al_2_O_3_ and characterized as light porous particles with large water absorbing ability, sewage sludge has potentials as high recycling value for construction industry. Researchers have investigated the use of sewage sludge ash (SSA) for various construction applications, such as components of bricks and tiles, raw material for cement production, aggregates of concrete and mortar, a synthesized component of lightweight materials, and substitutes for sand and/or cement in road pavement [2].

Among these applications, applying SSA to produce bricks and tiles has been a major area with promising potentials and has attracted much attention in academia. Tay [3] was one of the first pioneers to mix dry sewage sludge with clay to fabricate blocks, finding 40% dry sludge replacement a feasible solution. Tay and Goh [4] later found some blemishes appeared in the tiles with a high sludge replacement ratio. In order to better understand the properties of incinerated sewage sludge ash (ISSA) for possible construction use, Lin [5] studied the mineralogy and microstructures of SSA and found that the bulk density of the sintered monoliths reduced as the sintering temperature increased from 1050 to 1100 °C. Lin and Weng [6] added various percentages of SSA, ranging from 0% to 50% to clay in producing brick samples. After a series of tests, the results showed that an appropriate quantity of sewage sludge ash replacement was below 20%. Weng and Lin [7] later investigated SSA tiles further and discovered some major shortcomings of SSA tiles, including excessive water absorption, deficient abrasion, and extensive porosity. Many attempts have been made to resolve such shortcomings. Lin et al. [8] applied glaze with different colorants to ISSA biscuit tile specimens and found that glaze improved the bending strength, abrasion resistance, and acid-alkali resistance of tiles. Wiebusch and Seyfried [9], mixing sewage sludge with clay to manufacture bricks and tiles, noticed that different SSAs have different influences such as the ceramic qualities of bricks or tiles. Monteiro et al. [10] used SSA contents of 0, 3, 5, and 10 wt % to manufacture tiles at different kiln temperatures and found that the addition of 10 wt % SSA to the tiles improved their water absorption behavior but slightly reduced their bending strength. Zhou et al. [11] performed a series of formulation experiments and characterized the physical and chemical properties of tiles and found that the maximum quantity of sewage sludge used in the spilt tiles is 60%. Lin et al. [12] applied nano-SiO_2_ as an additive to tiles containing SSA. They proved that tile bending strength improved with increased nano-SiO_2_ amount. Moreover, Lin et al. [13] studied the influences of kiln temperature and the amount of nanomaterial additive on the properties of SSA tiles and concluded that tile bending strength increased with increasing temperature when kiln temperatures were lower than 1100 °C, while both bending strength and firing shrinkage decreased as temperature went beyond 1150 °C.

Glass has also become a useful additive for improving SSA tiles. Lin and Cheng [14] mixed clay and different amount of solar panel waste glass to manufacture eco-tiles, and used XRD (X-ray diffraction), FTIR (Fourier transform infrared spectroscopy), and SEM (scanning electron microscopy) to investigate the characteristics of the microstructures of the specimens. Kim et al. [15] used LCD (liquid crystal display) waste glass as a flux material to replace the traditional feldspar in the manufacture of the ceramic tile specimens. They found that the calcined tile body containing LCD waste glass had a dense microstructure and had positive influences on tile specimens such as water absorption and the thermal expansion coefficient. Rozenstrauha et al. [16] spotted dense glass-ceramics composite on SSA/glass ceramics at the temperature between 1120 and 1140 °C. The crystalline phases of quartz (SiO_2_), anorthite (CaAl_2_Si_2_O_8_), and hematite (Fe_2_O_3_) were observed by XRD analysis, and these mineralogical compositions were proved to improve compressive strength of the glass-ceramics specimens. Fan and Li demonstrated that the LCD waste glass as the fluxing agent and MgO as the modifying agent can be used as a raw material for producing insulating glass ceramics [17].

To further examine the properties of reclaimed tile with SSA and waste glass, this study probed the persistence of the tile in extreme environments. Industrialization has led to acid rain and other harmful pollutants in major metropolitan areas, and the effects of acid and pollutants on reclaimed tiles have not been carefully studied. The main purpose of this study was thus to perform acid-alkali resistance tests on reclaimed tiles to inspect the variations in tile properties caused by the amount of SSA/glass replacement. The goal was to obtain a better SSA/glass replacement ratio benefiting the manufacture of the reclaimed tile to meet more demanding requirements. Before the acid-alkali resistance tests, a series of tests to identify basic material attributes including thermal characteristics, physical and mechanical properties, and mineralogical features was performed. Discussion and explanations are made herein to link and justify acid-alkali resistance test results and the basic material attributes.

## 2. Materials and Methods

The clay used in this study was a gray-white powder with a specific gravity of 2.33. Its chemical compositions were O (45.02%), Si (23.52%), Al (14.11%), K (7.52%), and Ca (4.77%). The compositions were determined by energy dispersive X-ray analysis (EDXA) (Oxford’s Instrument, Abingdon, UK), which detects unique sets of peaks with different atomic structures on a sample’s X-ray emission spectrum.

The sewage sludge cakes were acquired from the Kaohsiung City wastewater treatment plant with a specific gravity of 2.33 and moisture content of 75%. These cakes were naturally dried for 1 to 2 days before initially incinerated in a kiln at 800 °C for 20 h. After kiln incineration, the cakes were ground into a powder to pass through #200 sieves by a multi-spinning powder-grinding machine. The chemical compositions of sewage sludge ash (SSA) were then analyzed with EDXA; the SSA was found to include O (50.34%), Si (37.86%), Ca (2.66%), Fe (2.95%), and Al (2.38%), as well as slight amounts of Mg, K, Cr, and Zn. Waste glass sample used in this study was acquired from a recycling company near Kaohsiung City and had a specific gravity of 2.39. The chemical compositions of the waste glass sample were found to be O (46.87%), Si (28.44%), Na (7.85%), and Ca (7.12%), and trace amounts of Mg, Al, and C were detected.

Before being sent for making specimens, the SSA and waste glass samples underwent a toxicity characteristic leaching procedure (TCLP), which is to ensure that the raw materials are free of toxic hazards. TCLP simulates the conditions that may be present in a landfill, where water may pass through the land-filled waste and travel into the groundwater carrying the soluble materials with it. The test was performed according to NIEA R201.14C [18] (National Institute of Environmental Analysis, Taipei, Taiwan), which is equivalent to EPA Test Method 1311 [19] (Environmental Protection Agency, Washington, DC, USA). TCLP test results showed that the leached metal concentrations of these materials complied with the requirements set by the Environmental Protection Agency of Taiwan, as shown in Table 1.

In this study, the amounts of clay replaced with waste glass were 0%, 10%, 20%, 40%, and 60% for the fabrication of reclaimed tile specimens with 10% SSA replacement. In the process of fabrication, clay, SSA, and different amounts of waste glass were uniformly mixed in a shaft clay mixer first, and the mixtures were kneaded with a de-airing vacuum pug mill to reduce extra interior pores. Afterwards, the well-kneaded mixtures were placed in a mold with the size of 12 × 6 × 1 cm^3^ and compressed by a pressing machine with a normal pressure of 34.32 ± 0.5 MPa to produce the specimens. These molded specimens were next placed in a wooden plate and dried down in room temperature. After drying, the specimens were left in a kiln at a temperature of 1000 °C. Kiln temperature was controlled at a rate of 2 °C/min increase. Then, the specimens were cooled to room temperature in the kiln to complete the fabrication. The tests conducted during the course of this study included tests of abrasion resistance, weight loss on ignition, shrinkage, and bending strength to determine the relevant physical and chemical properties of the reclaimed tile specimens based on the CNS (Chinese National Standard) 3299 standards [20], which are equivalent to ISO (the International Organization for Standardization) 10,545 standards [21]. Furthermore, a Shimadzu X-ray diffractometer (model XRD-6000) (Tokyo, Japan) was used for X-ray diffraction (XRD) analysis at a scan rate of 2°/min, a diffraction angle (2θ) of 10 to 60 degrees, an electric current of 30 mA, and an operating voltage of 40 kV. Scanning electron microscopy (SEM) equipment (model S-4700, Hitachi, Tokyo, Japan) was used to study the microstructure of the reclaimed tile specimens.

To perform the acid-alkali resistance tests, the reclaimed tiles specimens containing different amounts of waste glass were submerged in HCl and KOH solutions. The pH values of acidic and alkaline solutions were maintained at 4 and 10, respectively. The resistance tests were performed at room temperature, and the specimens were retained in the acidic and alkaline solutions for 1-, 3-, and 7-day periods. At the end of each experimental period, tests including porosity, weight measurement, bending strength, and X-ray diffraction (XRD) tests were carried out for the particular specimens.

## 3. Results and Discussion

### 3.1. Basic Material Properties

#### 3.1.1. TGA and DTA (Thermogravimetric Analysis and Differential Thermal Analysis)

This study used thermogravimetric analysis (TGA) and differential thermal analysis (DTA) to examine thermal properties of the clay, SSA, and waste glass as they change with temperature. The results could be used to explain how these raw materials behave during kilning.

**Figure 1:**
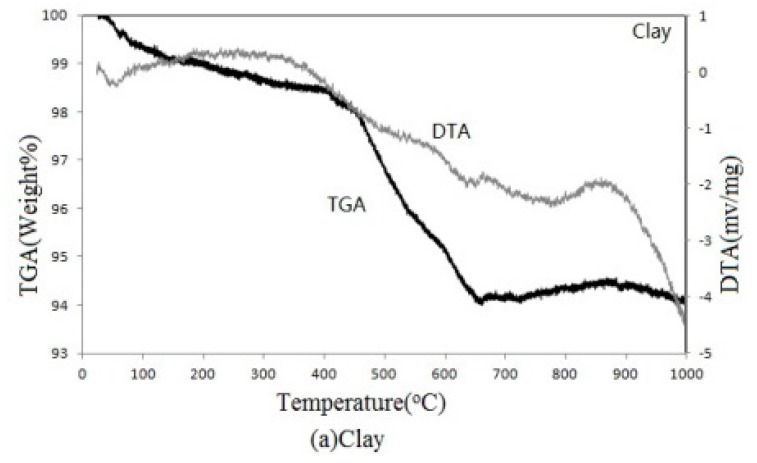

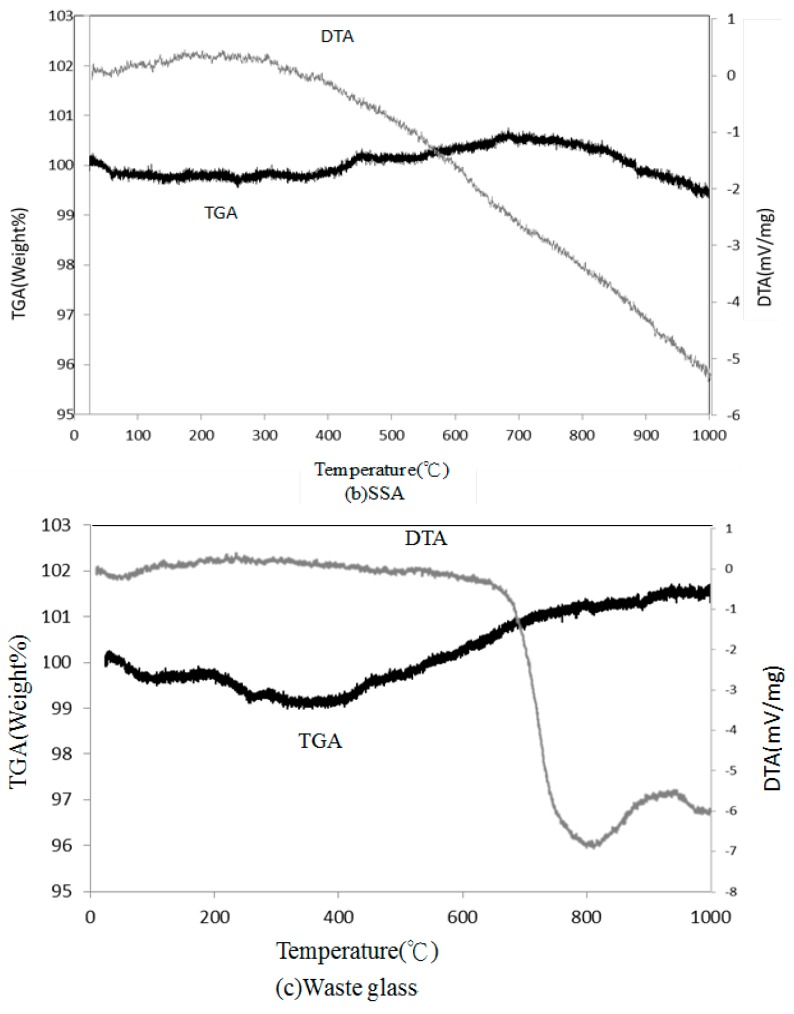
Thermogravimetric analysis (TGA) and differential thermal analysis (DTA): (**a**) clay; (**b**) SSA; (**c**) waste glass.

Figure 1a,c shows the results of the TGA and DTA. As shown in Figure 1a, the clay slowly absorbed heat and had gentle weight loss from its initial calcination process. Starting from 400 °C, the clay began a quick weight loss, accompanied by noticeable heat absorption. As the temperature reached beyond 640 °C, the weight of the clay was kept at about 94 wt %. It suggests that the clay sintering was completed when the temperature went higher than 700 °C. This result complied with the normal response to a change in temperature of typical clay minerals reported in an earlier study by Guggenheim and Van Groos [22].

Similar to the response of clay, heat absorption of the SSA was gentle at first and became harsher after 400 °C, according to the DTA curve of Figure 1b. However, different from clay, which continuously lost noticeable weight after 400 °C, SSA was observed to lose weight after 700 °C, but its magnitude was negligible compared with clay based on the TGA curve of Figure 1b. A previous incineration process could be the reason of such a steady thermogravimetric performance of SSA.

Different from the relative mild heat absorption of clay and SSA, which was gently triggered at an earlier stage, waste glass started action at 700 °C, with a more explosive style. Quick and massive heat absorption was spotted between the temperature of 700–800 °C, and somehow began releasing heat afterwards. Compared with clay, thermogravimetric loss of waste glass was considered mild and steady. TGA in Figure 1c shows that the waste glass slightly lost weight until 350 °C, for a total of less than 1%. It was figured that, before 350 °C, the organic matter in the waste glass was completely burned and that the inside structure of waste glass began to sinter as the temperature went beyond 350 °C. Then, after the temperature reached 700 °C, the glass began to melt and started bonding together, while massive heat absorption took place simultaneously.

The results of the TGA showed that both SSA (after incineration) and waste glass achieved better thermogravimetric stability than clay. When used as a clay replacement for manufacturing reclaimed tiles, both SSA and waste glass could maintain mass integrity and help to produce tiles with uniform quality. DTA shows that SSA and clay shared a similar calcination behavior throughout the entire kilning period. At the same time, the heat absorbing reaction of waste glass to cause a structural meltdown was delayed after 700 °C. This delayed heat absorbing feature of waste glass could be beneficial in allowing melted glass to fill in the voids induced by earlier calcination of SSA and clay, resulting in more compact tiles.

#### 3.1.2. Physical and Mechanical Analysis

Physical and mechanical tests conducted in this study included abrasion resistance, shrinkage, weight loss on ignition, water absorption, and bending strength, and the tests were performed according to the following CNS standards: the abrasion resistance test by CNS 3299-5 [20] equivalent to ISO 10545-5:1996 [21]—Ceramic tiles—Part 5: Determination of impact resistance by measurement of coefficient of restitution; the shrinkage test by CNS 3299-2 equivalent to ISO 10545-2:1995 [21]—Ceramic tiles—Part 2: Determination of dimensions and surface quality; the weight loss on ignition test by CNS 8436 M3081 [23] similar to ISO 11536:2015 [24] Iron ores: Determination of loss on ignition—Gravimetric method; the water absorption test by CNS 3299-3 [20] equivalent to ISO 10545-3:1995 [21]—Ceramic tiles—Part 3: Determination of water absorption, apparent porosity, apparent relative density and bulk density; and the bending strength test by CNS 3299-4 [20] equivalent to ISO 10545-4:1995 [21]—Ceramic tiles—Part 4: Determination of modulus of rupture and breaking strength. Table 2 shows the results of these tests. The abrasion resistance of the reclaimed tiles decreased with an increasing level of waste glass replacement. This implies that the use of waste glass could reduce the abrasion and improve the quality of reclaimed tiles. Simultaneously, the bending strength of the reclaimed tiles gradually increased. Moreover, the shrinkage of the reclaimed tiles increased, and the weight loss on ignition was reduced. These findings suggest that the waste glass particles were relatively large in size and resisted water absorption. Upon calcination at 1000 °C, low-viscosity liquid glass was produced and filled in the pores among the particles. This liquid glass then became bound with the viscous SSA generated during the same calcination process. As a result, better compactness was achieved in the bodies of the reclaimed tiles containing waste glass and SSA.

With the introduction of only 10% waste glass, the porosity, shTerinkage, weight loss on ignition, and water absorption of the reclaimed tile specimens showed only a slight change. However, when the level of waste glass replacement was increased to 20%, evident changes in these properties were observed. Moreover, the bending strengths of the reclaimed tiles began to decrease when the waste glass content was increased to above 20%. It is concluded here that 10% SSA replacement and waste glass content between 10% and 20% resulted in the best mechanical performance of the manufactured tile specimens.

#### 3.1.3. Mineralogical Analysis

**Figure 2:**
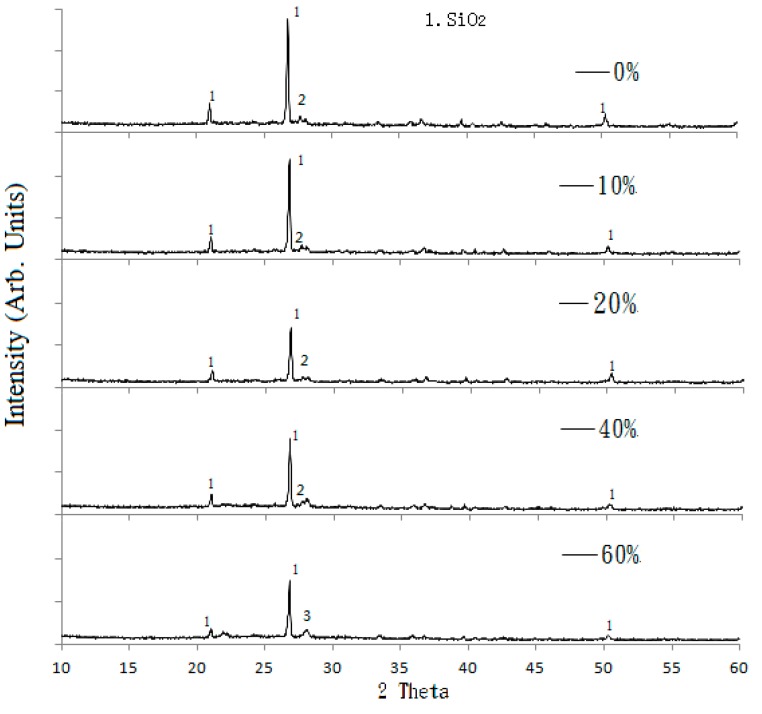
The XRD (X-ray diffraction) spectra of the reclaimed tile specimens, with 0%, 10%, 20%, 40%, and 60% waste glass replacement calcined at 1000 °C.

XRD analysis was used to observe the changes in the crystallinity of the reclaimed tile specimens. Figure 2 shows the XRD spectra for the reclaimed tile specimens with weight fractions of 0%, 10%, 20%, 40%, and 60% waste glass replacement calcined at 1000 °C. As shown in the figure, peaks associated with SiO_2_ crystal were clearly observed at all levels of waste glass replacement, suggesting that the presence of the waste glass did not impede crystallinity in the reclaimed tiles, although glass has been regarded as non-crystalline amorphous solid. However, the level of crystallinity of the SiO_2_ in the specimens weakened as the level of waste glass replacement increased because of the amorphous nature of glass. This suggests that the SiO_2_ from waste glass remained non-crystalline amorphous, but that the SiO_2_ in SSA and clay was instead converted into a crystalline form during calcination at 1000 °C.

**Figure 3:**
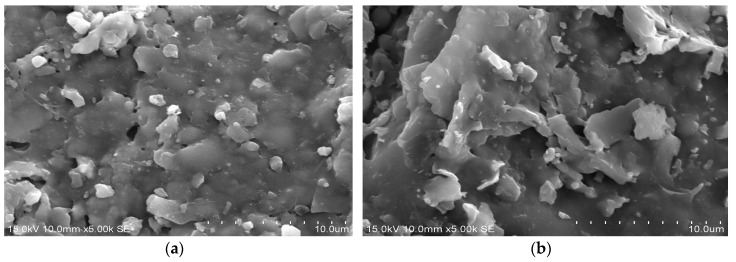

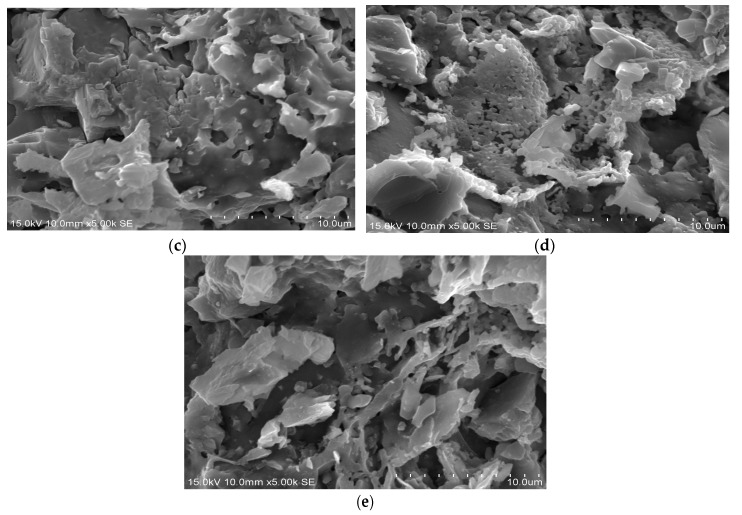
Scanning electron microscopy (SEM) images (5000×) of the reclaimed tile specimens with (**a**) 0% (W/O Waste Glass); (**b**) 10%; (**c**) 20%; (**d**) 40%; and (**e**) 60% waste glass replacement calcined at 1000 °C.

Figure 3a–e shows SEM images (magnification ratio at 5000 times, 5.00 k) of the reclaimed tile specimens with 0%, 10%, 20%, 40%, and 60% waste glass replacement calcined at 1000 °C.

Figure 3a, which is the SEM image of pure SSA reclaimed tile with 0% waste glass replacement, reveals a less compact structure with distinct particles and an uneven texture. Such a structure signifies the higher porous tile bodies. With the introduction of waste glass, silicate with a glassy structure formed inside the reclaimed tile specimens. Hence, the pores inside the tile bodies were gradually filled in, resulting in more compact tile bodies. Therefore, the physical and mechanical properties of the reclaimed tile specimens with various levels of waste glass replacement were improved.

### 3.2. Acid-Alkali Resistance Tests

**Figure 4:**
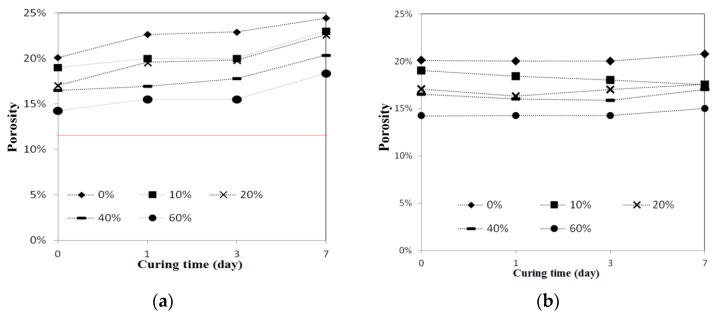
The acid-alkali resistance test results of porosity for the reclaimed tile specimens containing 0%, 10%, 20%, 40%, and 60% waste glass replacement calcined at temperature of 1000 °C when cured at different ages: (**a**) acidic solution and (**b**) alkaline solution.

**Figure 5:**
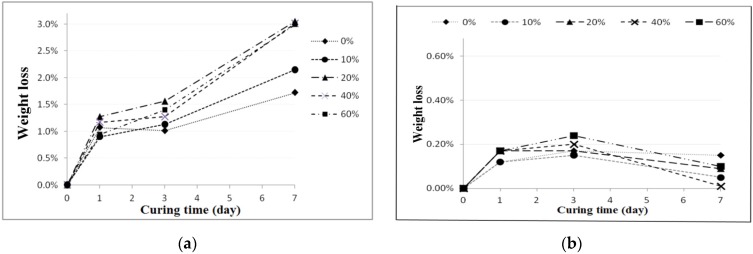
The acid-alkali resistance test results of weight loss for the reclaimed tile specimens containing 0%, 10%, 20%, 40%, and 60% waste glass replacement calcined at temperature of 1000 °C when cured at different ages: (**a**) acidic solution and (**b**) alkaline solution.

To examine reclaimed tile resistance in extreme acid and alkali environment, tile porosity change, weight loss, and bending strength tests were performed. These acid-alkali resistance tests were conducted at room temperature after leaving the specimens in the HCl (pH 4.0) and KOH (pH 10.0) solutions for curing ages from one, three, and seven days. Figure 4 shows the porosity change test on 0%, 10%, 20%, 40%, and 60% waste glass replacement over different curing times, and the test was again performed according to ISO 10545-3:1995 [21] specification for Ceramic tiles—Part 3: Determination of water absorption, apparent porosity, apparent relative density and bulk density. The result shows that tile porosity increased with a longer curing time in the acidic solution, while porosity had little change over time in the alkaline solution. Figure 5 shows conceivable weight loss on 0%, 10%, 20%, 40%, and 60% waste glass replacement when cured at different ages. Similar to porosity, weight loss increased with a longer curing time in the acidic solution, but weight loss did not change much in the alkaline solution. Furthermore, as waste glass replacement increased, the weight loss seemed to increase further.

**Figure 6:**
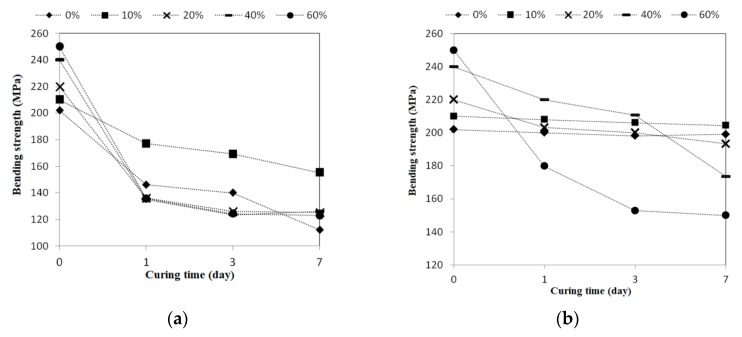
The acid-alkali resistance test results of bending strength for the reclaimed tile specimens containing 0%, 10%, 20%, 40%, and 60% waste glass replacement calcined at temperature of 1000 °C when cured at different ages: (**a**) alkaline solution; and (**b**) acidic solution.

Figure 6 shows the bending strength test results after various curing ages. In the acidic solution (as shown in Figure 6a), all tiles (0%, 10%, 20%, 40%, and 60%) had a significant bending strength reduction after one day, and their strengths had remained relatively steady afterwards with only minor decline until the end. In the alkaline solution (as shown in Figure 6b), the bending strengths of the tiles containing 60% and 40% waste glass were quickly reduced with a longer curing time in the acidic solution; on the other hand, the bending strength of those with lower waste glass replacements did not show any apparent change over time.

In general, the reclaimed tiles with waste glass had a better alkaline resistance than acidic resistance in all three categories of porosity, weight loss, and bending strength, except for the fact that the reclaimed tiles containing higher waste glass replacement had difficulty maintaining their bending strength in the alkaline environment.

With or without waste glass, the reclaimed tiles suffered considerable weight loss and increased porosity in the acidic solution, resulting in great bending strength reduction. One possible explanation is that the surface materials of alkaline dioxides on the tile specimens were able to react with the acid soluble salts and were rapidly decomposed with time, which directly caused the weight loss and porosity of the reclaimed tile specimens and also damaged the tile structure to reduce its bending strength. On the other hand, those reclaimed tiles without too much glass replacement performed much better in the alkaline solution. It is thought that, despite the fact that the materials in the tiles also reacted with alkaline, the chemical reaction happened to have positive effect on the tile and yielded a different result. The chemical reaction of tiles in the alkaline solution, SiO_2_ + KOH → K_2_SiO_3_↓ + H_2_O, produced a special compound K_2_SiO_3_, known as potassium silicate, which is usually characterized with good mechanical strength, high binding performance, and small porosity. Hence, with the production of the K_2_SiO_3_, the reclaimed tiles could withstand in the alkaline solution without increasing porosity and weight loss for a long period of time.

**Figure 7:**
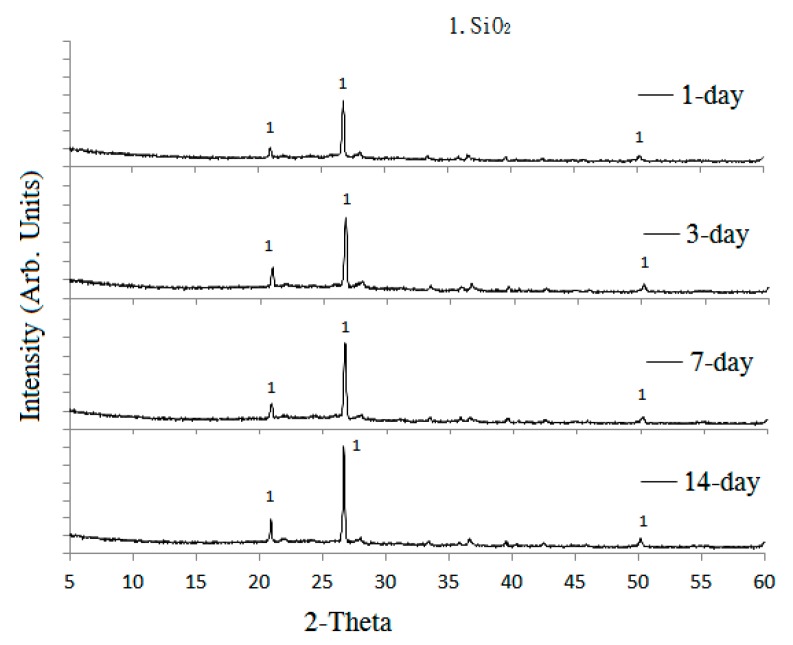
Results of XRD for the reclaimed tile specimens containing 10% SSA and 10% waste glass replacements when cured in the acidic solution at different ages.

To further examine how interior crystalline state change in the tile body during loss of resistance, XRD was again performed on the specimens after acidic curing. Figure 7 shows results of XRD for the reclaimed tile specimens containing 10% SSA and 10% waste glass replacements after being cured in the acidic solution for different ages, from 1 to 14 days. The results show that peak intensities of SiO_2_ in the specimens increased with a longer curing time in the acidic solution. This is an indication of increased crystalline formation on the tile surface. Based on the results of XRD analysis presented in Figure 2, as the non-crystalline amorphous nature of waste glass weakens the peak intensities of SiO_2_ in XRD, the resurgence of SiO_2_ intensities with a prolonged curing time indicated the diminishing of the waste glass. It is thought that waste glass replacement in the reclaimed tiles had gradually decomposed into the acidic solution after a long curing time since waste glass has relatedly low resistance to acid compared with clay. The loss of the waste glass structure to reinforce the tile body also means the loss of bending strength as well as increased weight loss and porosity, which has been verified in the acid-alkali resistance tests of this study.

## 4. Conclusions

In this study, the possible application of using waste glass and sewage sludge ash (SSA) to replace part of the clay in the manufacture of reclaimed tiles was further investigated. Test results performed in the normal environment show that the main chemical components of the waste glass and SSA were silicon dioxide and alumina, respectively. The addition of waste glass to the reclaimed tile specimens decreased the porosity and water absorption of the tiles and enhanced their bending strength. Moreover, after the completion of the calcination of the reclaimed tile specimens containing waste glass and SSA, the weight loss of the tile bodies was apparently reduced. When 10% SSA replacement was applied in the reclaimed tile specimens, increasing the waste glass content resulted in higher shrinkage and reduced weight loss on ignition in the bodies of the reclaimed tiles. Furthermore, the XRD spectra show that the SiO_2_ peak intensity decreased with an increasing level of waste glass replacement. The Al_2_O_3_ content played an important role in crystallization in the bodies of the reclaimed tiles. These findings suggest that the production of crystalline structures in the tile bodies might be affected by the waste glass content. The results of this study indicate that, for reclaimed tile specimens with 10% SSA replacement, the optimal level of waste glass replacement is between 10% and 20%. Acid-alkali resistance tests were performed on the reclaimed tiles. It was found that the reclaimed tiles performed well in the acid-alkali resistance tests, especially in the alkali resistance test. Hence, it is suggested that the reclaimed tiles can be applied to the alkaline environment. Furthermore, extra weight loss of the reclaimed tiles was noticed in the acidic environment. The weight loss increased with the increased amount of the waste glass replacement. The reduction in bending strength of the reclaimed tiles was larger in the acidic environment than that in the alkaline environment. The amount of the waste glass replacement had an impact on the bending strength of the reclaimed tiles. Based on the results of this study, it is not recommended that reclaimed tiles be placed in acidic environments over a long period of time.

## Figures and Tables

**Table 1:** The test results from the toxicity characteristic leaching procedure (TCLP).

Heavy Metal (mg/L)	As	Pb	Cu	Cd	Zn	Cr	Hg	Cr^6+^	Se	Ba
SSA	0.42	<0.2	7.94	<0.2	11.3	<0.2	ND	ND	ND	<0.2
Waste Glass	ND	0.38	0.13	ND	ND	0.06	ND	ND	ND	1.05
Standard	5	5	15	1	25	5	0.2	2.5	1	100

**Table 2:** Physical and mechanical performances of the reclaimed tile specimens.

Test	Waste Glass Content (%)					Tendency
	0%	10%	20%	40%	60%	
Abrasion (g)	0.042	0.031	0.025	0.021	0.023	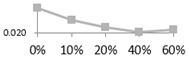
Shrinkage (%)	6	5.5	6.1	6.5	8.4	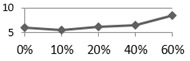
Weight loss on ignition (%)	5.5	5	4.6	3.5	2.8	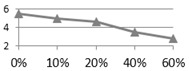
Water absorption (%)	17.1	16.8	15.3	14.3	14	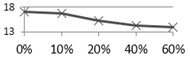
Bending strength (MPa)	232	251	270	268	272